# Deep sequencing of mitochondrial DNA and characterization of a novel *POLG* mutation in a patient with arPEO

**DOI:** 10.1212/NXG.0000000000000391

**Published:** 2020-01-10

**Authors:** Carola Hedberg-Oldfors, Bertil Macao, Swaraj Basu, Christopher Lindberg, Bradley Peter, Direnis Erdinc, Jay P. Uhler, Erik Larsson, Maria Falkenberg, Anders Oldfors

**Affiliations:** From the Department of Pathology and Genetics (C.H.-O., A.O.) and Medical Biochemistry and Cell Biology (B.M., S.B., B.P., D.E., J.P.U., E.L., M.F.), University of Gothenburg; and Neuromuscular Centre (C.L.), Department of Neurology, Sahlgrenska University Hospital, Gothenburg, Sweden.

## Abstract

**Objective:**

To determine the pathogenicity of a novel *POLG* mutation in a man with late-onset autosomal recessive progressive external ophthalmoplegia using clinical, molecular, and biochemical analyses.

**Methods:**

A multipronged approach with detailed neurologic examinations, muscle biopsy analyses, molecular genetic studies, and in vitro biochemical characterization.

**Results:**

The patient had slowly progressive bilateral ptosis and severely reduced horizontal and vertical gaze. Muscle biopsy showed slight variability in muscle fiber size, scattered ragged red fibers, and partial cytochrome c oxidase deficiency. Biallelic mutations were identified in the *POLG* gene encoding the catalytic A subunit of POLγ. One allele carried a novel mutation in the exonuclease domain (c.590T>C; p.F197S), and the other had a previously characterized null mutation in the polymerase domain (c.2740A>C; p.T914P). Biochemical characterization revealed that the novel F197S mutant protein had reduced exonuclease and DNA polymerase activities and confirmed that T914P was inactive. By deep sequencing of mitochondrial DNA (mtDNA) extracted from muscle, multiple large-scale rearrangements were mapped and quantified.

**Conclusions:**

The patient's phenotype was caused by biallelic *POLG* mutations, resulting in one inactive POLγA protein (T914P) and one with decreased polymerase and exonuclease activity (F197S). The reduction in polymerase activity explains the presence of multiple pathogenic large-scale deletions in the patient's mtDNA.

Cells contain thousands of mitochondrial DNA (mtDNA) molecules that are maintained by a dedicated mitochondrial replication machinery encoded by nuclear genes.^1,2^ mtDNA is replicated by POLγ, a trimeric protein comprising one POLγA catalytic subunit and a dimer of the processivity factor POLγB.^1^

Mutations in POLγA are one of the most frequent causes of mitochondrial disease, which is characterized by insufficiency in oxidative phosphorylation. Pathogenic POLγA mutations are associated with the accumulation of secondary mtDNA mutations and/or progressive mtDNA depletion due to errors in DNA replication.^3–6^ A common clinical manifestation of POLγA-related diseases is progressive external ophthalmoplegia (PEO), a disorder involving progressive weakening of the extraocular muscles that is frequently combined with symptoms from other parts of the neuromuscular and nervous system.

Here, we report a patient with PEO carrying biallelic POLγA mutations. One allele carries the previously characterized T914P null mutation in the polymerase domain,^7^ and the other allele carries the F197S mutation in the 3′-5′ exonuclease domain, which is described here for the first time. To gain a molecular understanding of the patient's phenotype, we performed clinical and laboratory investigations and characterized the biochemical properties of the mutant proteins.

## Methods

### Case report

The patient investigated is a man aged 69 years. His father had kidney failure of unknown reason, and his mother died at age 92 years. Neither of them had ptosis, hearing impairment, or muscle complaints. He has a healthy 68-year-old brother and 2 healthy sons aged 33 years and 37 years. The patient had normal motor milestones as a child, did his military service at age 20 years, and has always been physically active. He has no history of migraines, visual or hearing impairment, and cardiac or cognitive complaints or symptoms. At age 55 years, he noted slowly progressive bilateral ptosis and also a limitation in both horizontal and vertical gaze. Since age 65 years, he has complete horizontal ophthalmoplegia, no upward gaze whatsoever, but has limited downward gaze. He has been bilaterally operated for ptosis. A complete clinical neurologic examination was otherwise normal except a slight bilateral sensory-neuronal hearing impairment thought to be a cochlear affliction due to his mitochondrial disorder. Cardiac examination, including ECG and 24-hour Holter ECG, was normal. Brain MRI showed slight atrophy of the mesencephalon, pedunculus cerebelli superior, and frontotemporal parts of the brain. Clinically, he has no cognitive impairment, although no formal cognitive investigation has been performed. Serum creatine kinase, troponin T, Nt-pro-BNP, creatinine, cystatin C, and thyroid function tests were normal as was CSF examination.

### Morphologic analysis

Open skeletal muscle biopsy from the deltoid muscle was performed at age 66 years. Specimens were snap frozen for cryostat sectioning and histochemistry. Standard techniques were applied for enzyme histochemistry.^8^

### Molecular genetic analysis

Exome sequencing (ES) was performed on blood DNA using the SureSelectXT Human All Exon kit v6 (Agilent Technologies, Santa Clara, CA) and sequenced on the HiSeq2500 platform (Illumina, Inc., San Diego, CA). The paired-end reads were aligned to the reference genome (hg19) using the CLC Biomedical Genomics workbench (QIAGEN GmbH, Hilden, Germany). Data were analyzed with Ingenuity Variant Analysis (QIAGEN). Candidate genes associated with myopathy were analyzed for biologically relevant variants that were predicted to be damaging using SIFT algorithm and PolyPhen2 and were not common in the human population (minor allele frequency below 1%).

### Analysis of mtDNA deletions using deep sequencing

DNA was isolated from muscle biopsies from the patient and 2 age-matched control individuals using standard protocols. DNA was subjected to whole-genome sequencing using the TruSeq PCR free library preparation kit (Illumina), and the Illumina HiSeq X platform was used for sequencing (Illumina). A previously described pipeline was used to align reads to nuclear chromosomes (hg19 assembly) and the mitochondrial genome (revised Cambridge Reference Sequence assembly, NC_012920.1) to identify deletions and duplications.^9^ We observed a chrM coverage depth of 106,170 for the patient (11,777,135 reads), whereas the controls were 54,426 and 101,942, respectively (6,092,978 and 11,263,816 reads). Gapped alignments, indicative of deletions/duplications, were clustered and visualized.^9^ Heteroplasmy levels for individual deletions/duplications were estimated by comparing the number of reads supporting the corresponding breakpoints to the total number of reads overlapping the breakpoints (including wild type) after removal of PCR duplicates.

### Mutagenesis and protein purification

The mutant POLγA variants were generated with the QuikChange Lightning Site-Directed Mutagenesis Kit (Agilent Technologies) and confirmed by sequencing (Eurofins MWG Operon, Ebersberg, Germany). Recombinant 6× His-tagged TWINKLE, POLγB, and the POLγA versions were expressed in baculovirus-infected Sf9 cells,^10^ purified over His-Select Nickel Affinity Gel (Sigma-Aldrich AB, Stockholm, Sweden) and HiTrap Heparin HP, followed by HiTrap SP HP, HiTrap Q HP, or both columns. *E. coli* BL21(DE3)-expressed mitochondrial single-stranded DNA binding protein (mtSSB) was purified over DEAE Sepharose Fast Flow, HiTrap Heparin HP, HiTrap SP HP, and HiLoad Superdex 200 (GE Healthcare, Uppsala, Sweden).

### Electrophoretic mobility shift assay

A primed template, consisting of a ^32^P 5′-labeled 21-nt oligonucleotide (5′- GCGGTCGAGTCTAGAGGAGCC-3′) hybridized to a 36-nt oligonucleotide (5′GACTACGTCTATCCGGGCTCCTCTAGACTCGACCGC-3′) (figure 2A, lower panel), was used to examine POLγ-DNA binding affinity. Fifteen-microliter reactions contained 10 fmol DNA template, 25 mM Tris-HCl (pH 7.8), 1 mM dithiothreitol, 0.1 mg/mL bovine serum albumin, 10% glycerol, 0.3 mM dideoxyguanosine triphosphate, 3 mM deoxycytidine triphosphate, and the holoenzyme (POLγA/POLγB complex). Reactions were incubated for 10 minutes at ambient temperature, separated on 6% native polyacrylamide gels in 0.5 × TBE for 30 minutes at 180 V, and visualized by autoradiography.

### 3′-5′ exonuclease activity assay

A ^32^P-labeled 32-nt oligonucleotide (5′-CTATCTCAGCGATCTGTCTATTTCGTTCATCG3′) was annealed to single-stranded pBluescript SK+ plasmid, creating a 31-bp double-stranded DNA (dsDNA) region with a 3′-end one-nucleotide mismatch (figure 2D, lower panel). Reactions (20 µL) were performed using the same buffer conditions as above (but with 10 mM MgCl_2_) using 10 fmol DNA, but without any deoxynucleotide triphosphates to promote exonuclease activity. POLγA (150 fmol) and POLγB (300 fmol, dimeric) were added. Products were run in 7 M urea/20% polyacrylamide gels and visualized by autoradiography.

### DNA synthesis on a single-stranded DNA template

A ^32^P-labeled 32-nt oligonucleotide (5′-CTATCTCAGCGATCTGTCTATTTCGTTCATCC- 3′) was hybridized to single-stranded pBluescript SK(+) plasmid. Reactions (20 µL) contained 10 fmol DNA template, 10 μM deoxynucleotide triphosphates, 25 mM Tris-HCl (pH 7.8), 1 mM dithiothreitol, 10 mM MgCl_2_, 0.1 mg/mL bovine serum albumin, 150 fmol POLγA, 300 fmol POLγB (dimeric), and 2.4 pmol mtSSB (tetrameric). Reactions were incubated at 37°C and stopped with 6 µL stop buffer (90 mM EDTA, 6% sodium dodecyl sulfate, 30% glycerol, 0.025% bromophenol blue, and 0.025% xylene cyanol). Products were analyzed on 0.8% agarose gels in 1 × TBE buffer and visualized by autoradiography.

### DNA synthesis on a dsDNA template

A 70-nt oligonucleotide (5′-T_42_ATCTCAGCGATCTGTCTATTTCGTTCAT-3′) was annealed to single-stranded pBluescript SK(+) followed by one cycle of polymerization using KOD polymerase (Merck Chemicals and Life Science AB, Stockholm, Sweden) to produce a ∼3-kb double-stranded template with a preformed replication fork. Reactions (20 µL) contained 10 fmol DNA, 10 µM deoxynucleotide triphosphates, 4 mM adenosine triphosphate, 2 μCi α-^32^P-deoxycytidine triphosphate, 4 pmol mtSSB (tetrameric), 100 fmol of TWINKLE (hexameric), and 250 fmol POLγA in complex with POLγB (dimeric) in the same buffer conditions as above. Reactions were incubated at 37°C and stopped with 6 µL alkaline stop buffer (18% [wt/vol] Ficoll, 300 mM NaOH, 60 mM EDTA [pH8], 0.15% [wt/vol] Bromocresol green, and 0.35% [wt/vol] xylene cyanol). Products were run in 0.8% alkaline agarose gels and visualized by autoradiography.^10^

### Standard protocol approvals, registrations, and patient consents

This study was approved by the local ethics committee. The study complied with the Declaration of Helsinki, and informed consent was obtained from the patient.

### Data availability

Data are available from the corresponding authors on reasonable request.

## Results

### Morphologic analyses demonstrate mitochondrial myopathy

**Figure 1:**
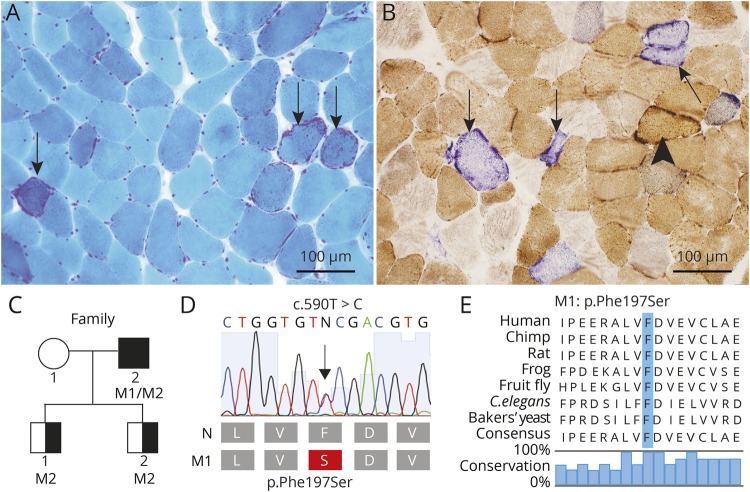
Morphologic analysis, pedigree, and molecular genetics (A) The muscle biopsy from the patient showed slight variability in muscle fiber size and scattered ragged red fibers (arrows) (Gomori trichrome staining). (B) Several fibers show cytochrome c oxidase (COX) deficiency with normal succinate dehydrogenase (SDH) activity (blue fibers, arrows), whereas a few fibers with mitochondrial proliferation show no COX deficiency (arrowhead) (combined enzyme histochemical staining of COX and SDH). (C) Pedigree of the family: filled symbol indicates affected individual with biallelic variants in *POLG*, and half-filled symbols indicate healthy heterozygous carriers. M1 indicates the novel variant c.590T>C; p.(F197S) and M2 c.2740A>C; p.(T914P) in *POLG* (NM_002693.2). (D) Illustration of the novel c.590T>C. p.(F197S) variant in *POLG*, with chromatogram. (E) Illustration showing the evolutionary conservation of amino acids. Blue bar indicates the mutated residue (p.Phe197Ser).

A muscle biopsy from the deltoid muscle at age 66 years showed slight variability in muscle fiber size, scattered ragged red fibers, and 10%–20% fibers with cytochrome c oxidase deficiency (figure 1, A and B).

### Molecular genetic analysis identifies *POLG* variants

ES revealed that the patient had 2 potentially pathogenic heterozygous missense mutations in the gene coding for the catalytic subunit of DNA polymerase gamma (*POLG*), the novel c.590T>C p.(F197S), and the previously described c.2740A>C; p.(T914P) (NM_002693.2) (figure 1, C and D). The variants were not common in the human population and affected conserved amino acids among species (figure 1E). Genetic analysis of the sons revealed that both were heterozygous carriers for the c.2740A>C; p.(T914P) variant (figure 1C), demonstrating that the mutations are located on different alleles.

### The F197S mutant has reduced 3′-5′ exonuclease activity

**Figure 2:**
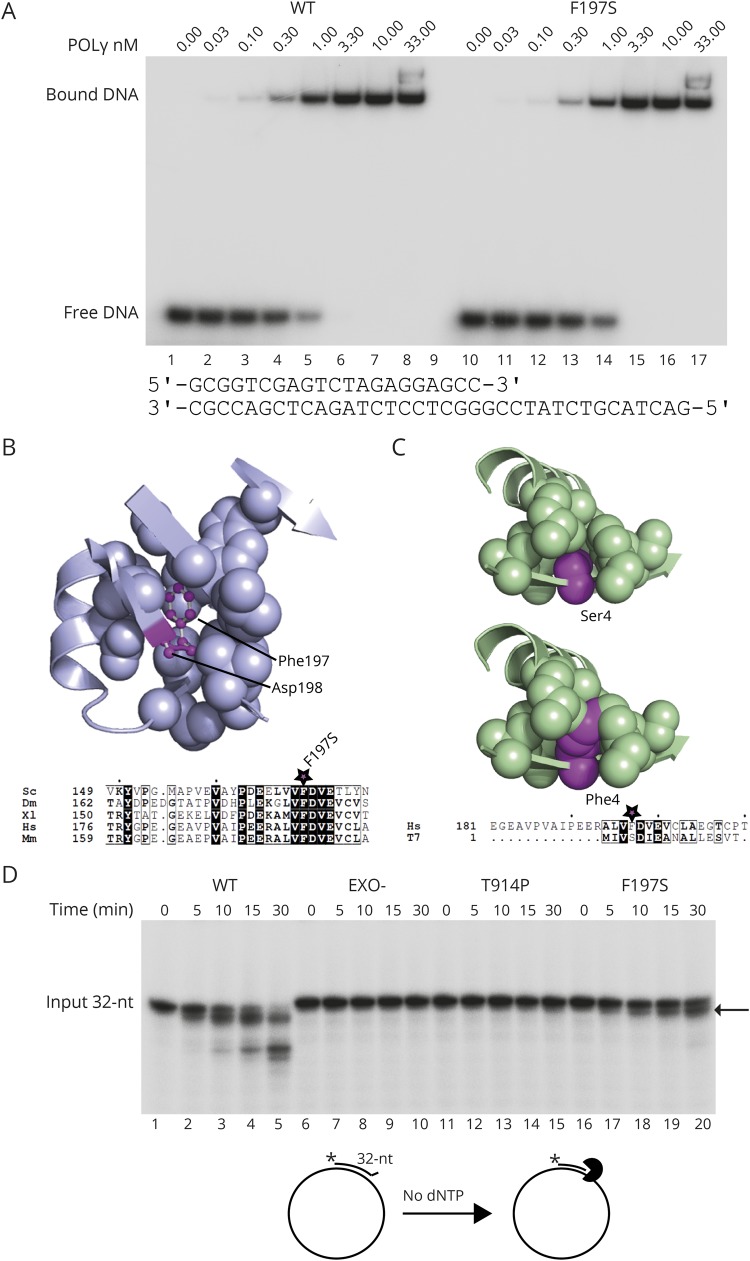
The F197S point mutation reduces 3′-5′ exonuclease activity (A) The F197S mutant has WT-like DNA binding affinity. DNA affinity of POLγ to a primer template (lower panel) was examined by electrophoretic mobility shift assay. Each reaction contained 10 fmol of DNA template and increasing (indicated) amounts of POLγ holoenzyme in a final volume of 15 µL. The reactions were run on 6% native polyacrylamide gels in 0.5 × TBE for 30 minutes at 180 V and visualized by autoradiography. No major differences in binding affinity between WT and F197S were observed. (B) The F197S point mutation disrupts the architecture of the exonuclease pocket. F197 is located in a large hydrophobic pocket directly adjacent to the catalytically essential D198 (upper panel; PDB ID: 3IKM). This residue is strongly conserved among members of the DNA polymerase A family (lower panel; Sc = *Saccharomyces cerevisiae*; Dm = *Drosophila melanogaster*; Xl = *Xenopus laevis*; Hs = *Homo sapiens*; Mm = *Mus musculus*). (C) The T7 exonuclease pocket is substantially smaller to accommodate a serine residue at the position equivalent to F197. Mutation of this residue to phenylalanine results in steric clashes, which would likely destabilize the pocket (PDB ID: 1T7P; Hs = *Homo sapiens*; T7 = bacteriophage T7). (D) A 5′-labeled (^32^P) primer with a 3′-mismatch was annealed to a circular single-stranded plasmid (lower panel to the left). POLγA with 3′-5′ exonuclease activity will, in the absence of dNTP, hydrolyze DNA into shorter fragments. As expected, WT degraded the primer (lanes 1–5), whereas EXO- could not due to lack of exonuclease activity (lanes 6–10). T914P showed no activity. The F197S mutant had weak exonuclease activity, only able to hydrolyze a few nucleotides under these conditions. The arrow indicates the partially hydrolyzed primer.

To further understand the molecular consequences of the mutations, we expressed and purified WT, F197S, and T914P POLγA for biochemical analysis. We previously showed that T914P had no DNA binding affinity.^7^ Given the patient's relatively mild condition, we predicted the F197S mutation to have no impact on DNA binding affinity, which we confirmed using electrophoretic mobility shift assay (figure 2A).

We next considered whether the Phe-197-Ser substitution affected 3′-5′ exonuclease activity. F197 is strongly conserved in the DNA polymerase A family across vertebrates and invertebrates (figure 2B, lower panel). In the 3′-5′ exonuclease domain, the F197 residue is located within a large hydrophobic pocket where it forms numerous van der Waals interactions with the surrounding residues (figure 2B, upper panel). Substitution of the bulky and hydrophobic phenylalanine with the smaller and polar serine would likely cause partial collapse of this pocket and local structural distortions. This may induce a misalignment of the neighboring D198 residue and subsequent reduction in its ability to bind a catalytically essential Mg^2+^ ion. Of interest, the closely related T7 DNA polymerase carries a serine at this position (figure 2C, lower panel) arguing that this amino acid change might not provoke structural or functional changes. Structural analysis, however, revealed that the exonuclease pocket is substantially smaller and that the presence of a phenylalanine would induce severe steric clashes and likely destabilize the exonuclease domain in T7 DNA polymerase (figure 2C, upper panel).

Despite being tolerated in T7 DNA polymerase, we decided to test whether the serine affects the 3′-5′ exonuclease activity of POLγA in vitro. A double-stranded template with a one-nt 3′-flap (figure 2D, lower panel) was incubated with the WT or mutant POLγ variants including a previously characterized exonuclease-deficient mutant D274A (herein EXO-) for comparison.^11^ As expected, WT POLγ was able to hydrolyze the primer (figure 2D, lanes 1–5), whereas EXO- was unable to degrade the primer (figure 2D, lanes 6–10). F197S was able to degrade the primer, however, to a much lower extent than WT. This demonstrates that in POLγA, unlike the related T7 DNA polymerases, a serine at this position is not well tolerated (figure 2D, lanes 16-20). No 3′-5′ exonuclease activity from T914P was observed (figure 2D, lanes 11–15), likely due to the lack of DNA binding rather than an enzymatic effect of the mutation.^7^

The exonuclease-deficient POLγ (EXO-) in mice causes a 5-fold increase in mtDNA point mutations,^12^ prompting us to investigate the mutation load of the patient's mtDNA. We isolated total muscle DNA from the patient and performed deep DNA sequencing. We identified a slight, but significant, increase in mtDNA point mutations in the patient compared with age-matched controls (table), consistent with our in vitro findings. However, the level of heteroplasmy is likely too low to by itself explain the disease phenotype in the patient.

**Table:**
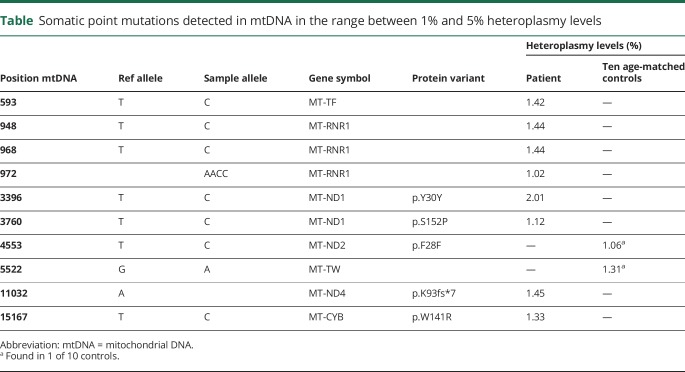
Somatic point mutations detected in mtDNA in the range between 1% and 5% heteroplasmy levels

### The F197S mutation affects DNA polymerase activity

**Figure 3:**
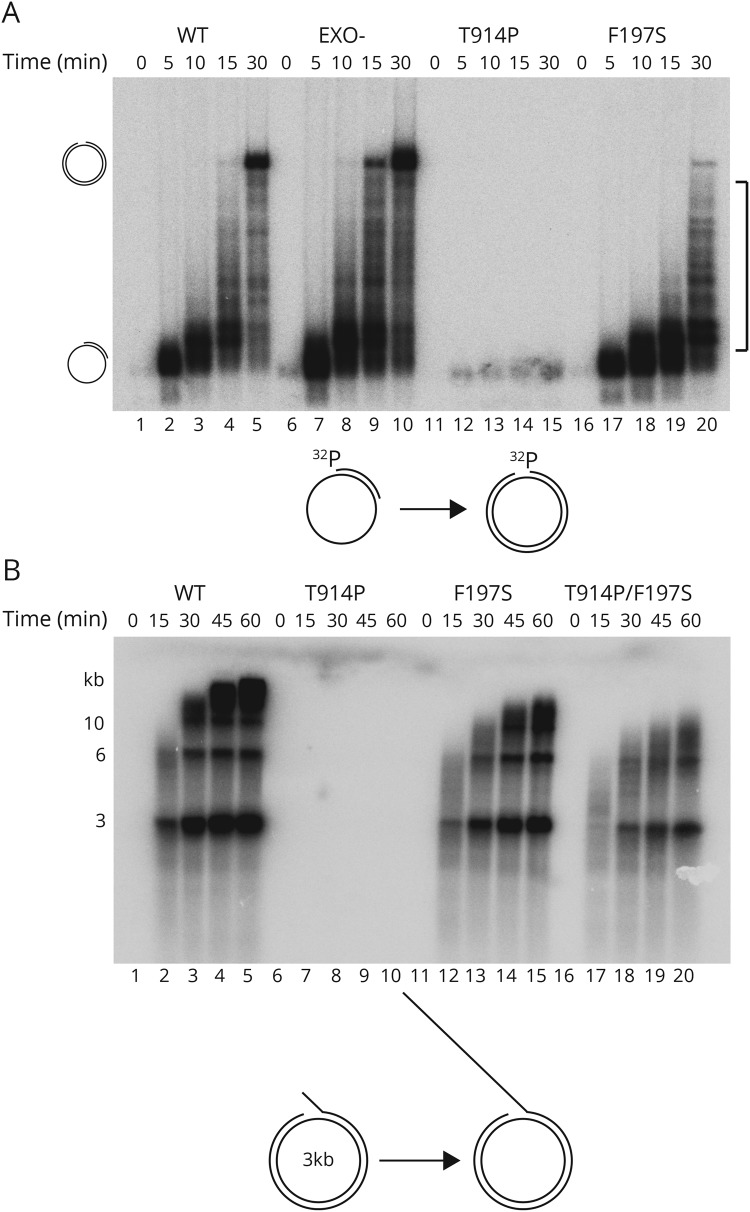
The F197S mutation affects DNA polymerase activity (A) DNA synthesis reaction on single-stranded DNA, where POLγA has the possibility to synthesize ≈3,000 nt. WT (lanes 1–5) and EXO- (lanes 6–10) POLγA displayed efficient DNA synthesis. F197S (lanes 16–20) was considerably slower, and T914P (lanes 11–15) was enzymatically dead. The squared bracket shows the positions of disrupted polymerization products. Below: A cartoon showing the template to the left and the fully synthesized product to the right. (B) DNA synthesis reaction on double-stranded DNA, where the replisome has the possibility to produce very long DNA products (>20 kb). The replisome, in the presence of F197S, was considerably slower than in the presence of WT. The T914P mutant was not able to support DNA synthesis. Mixing T914P and F197S (1:1 ratio) together to mimic the patient situation caused an apparent decrease in replication efficiency because only half of the total amount of polymerase (F197S) is active (lanes 16–20).

We next tested the polymerase activities of the mutants. First, we performed a DNA synthesis assay using a circular single-stranded DNA (ssDNA) template (figure 3A, lower panel). Because the F197S mutation is located in the 3′-5′ exonuclease domain, we also included EXO- for comparison. We found that F197S was active as a polymerase, albeit with strongly diminished activity compared with WT and EXO- (figure 3A, lanes 16–20 vs 1–5 and 6–10, respectively). The speed of replication was slower, generating less full-length product due to increased stalling. T914P was unable to support DNA synthesis (figure 3A, lanes 11–15).^7^

We also examined DNA synthesis in the context of the replisome (i.e., in the presence of the TWINKLE helicase and mtSSB) using a circular dsDNA template containing a preformed replication fork (figure 3B, lower panel). Here, F197S was able to synthesize long stretches of DNA, although it was not as efficient as WT (figure 3B, compare lanes 11–15 and 1–5). T914P was not able to support DNA synthesis (figure 3A, lanes 11–15 and 3B lanes 6–10).^7^ To mimic the in vivo situation (of the patient), a reaction was included where both disease-associated mutants were mixed at a 1:1 ratio, but keeping the same total amount of protein. This decreased the efficiency of replication further due to only half the amount of POLγA (F197S) being functional (figure 3B, lanes 16–20 vs 11–15).

### The F197S mutant causes multiple deletions and duplications in vivo

**Figure 4:**
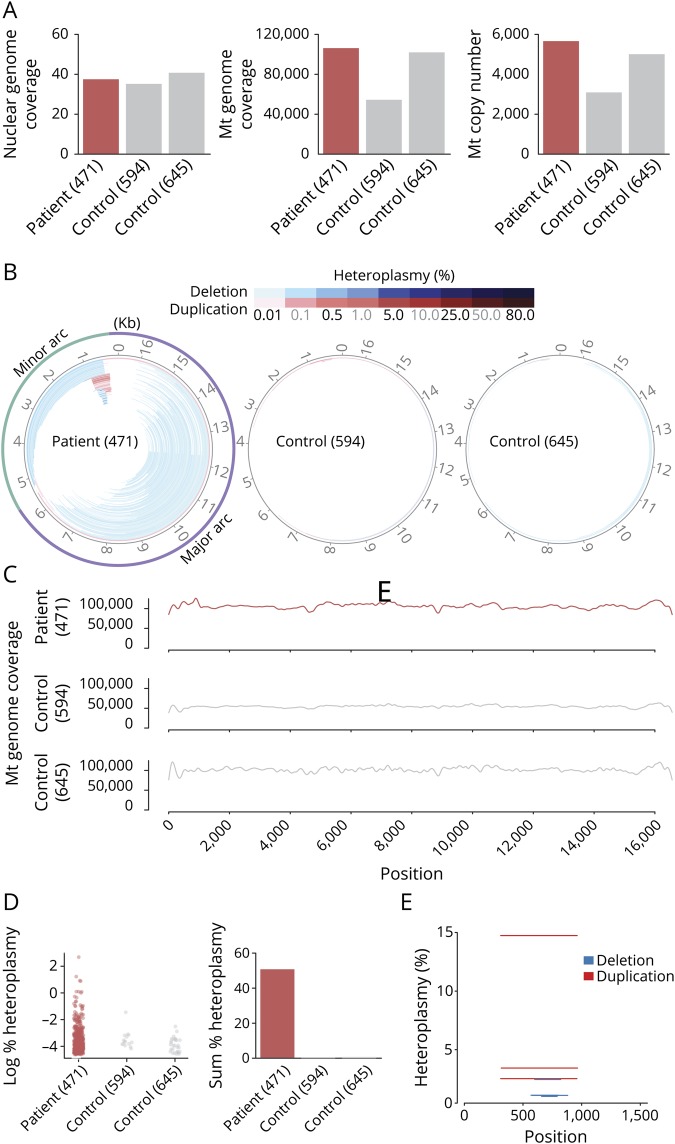
General statistics and distribution of breakpoints in the patient and control samples (A) Total coverage depth in the nuclear and mitochondrial genome of the samples along with estimated mitochondrial copy number. (B) Mitochondrial DNA (mtDNA) deletions (blue) and duplications (red) predicted from the sequencing data in the patient and control samples where the color intensity reflects the heteroplasmy level. Here, deleted regions are those showing gapped alignment of reads on the circular genome. Duplications are a gapped alignment indicating deletion of a genome fragment with an origin of replication, which is not plausible for a functioning mtDNA molecule. Thus, for duplications, the remaining fragment on the circular genome is considered as duplicated. (C) Coverage depth of sequencing reads along the mitochondrial genome in the patient and control samples. (D) Distribution of heteroplasmy for the detected deletions and duplications and the sum of the heteroplasmy levels for all deletions and duplications in the given samples. (E) Position and heteroplasmy levels of the deletions/duplications detected above 1% threshold heteroplasmy in the patient sample (none detected in the controls).

Disease-causing mutations in POLγ are known to cause mtDNA depletion and/or multiple mtDNA rearrangements. Based on the deep sequencing of isolated total muscle DNA, we found that mtDNA copy number in the patient was comparable to two controls, demonstrating that the patient does not exhibit mtDNA depletion (figure 4, A and C). However, the patient did have multiple deletions in both the major and minor arc (figure 4B). The sum heteroplasmy for the deletions and duplications amounted to 50.7%, which likely explains the patient's phenotype (figure 4D). Of interest, we also found a strong hotspot region for duplications spanning over the promoter region (position 300 nt–1,000 nt) where the top 3 candidate duplications were closely spaced (positions: 310–962, 310–960, and 315–965) (figure 4E) accounting for 21% heteroplasmy (14.7, 2.5, and 3.4% individually) of all deletions/duplications detected.

## Discussion

We describe a patient with myopathy, PEO, and ptosis associated with biallelic *POLG* mutations. By identification of mitochondrial myopathy caused by multiple large-scale mtDNA rearrangements and in vitro functional analysis of the identified *POLG* variants, we can explain several of the different molecular events leading to the disease and thus functionally link the primary genetic defect to the clinical phenotype.

*POLG* mutations are the most common cause of mitochondrial disease and are associated with a large variety of clinical phenotypes ranging from severe infantile neurodegenerative disorder with liver disease to late onset PEO with minor or no other symptoms.^13^ Our patient had a mild phenotype with ptosis and ophthalmoplegia as his main symptoms, requiring surgery for ptosis several years before his genetic diagnosis. His hearing deficit was probably due to the *POLG*-associated mitochondrial disease because hearing loss is a characteristic finding in patients with PEO, especially autosomal dominant PEO. Whether the brain atrophy was part of the syndrome has not been definitely established, but brain atrophy has been found in 28% of patients with *POLG*-associated epilepsy.^14^

Why some patients with mitochondrial disease develop PEO is not completely clear, partly because extraocular muscles are relatively inaccessible. However, MRI studies have demonstrated that extraocular muscles are atrophic in patients with PEO.^15,16^ The reason for this vulnerability has been speculated to depend on the distinct physiologic characteristics of extraocular muscles.^17^ They are very rich in mitochondria and are highly dependent on oxidative phosphorylation for endurance despite their composition of mainly fast-twitch fibers, and they develop age-related mitochondrial changes with multiple deletions at a considerably higher rate than limb muscles. It is therefore likely that they accumulate multiple deletions secondary to POLγA dysfunction at a higher rate than limb muscles, which then leads to atrophy and muscle fiber loss.

Our biochemical analysis of the mutant proteins showed that T914P had no activities, consistent with its previous characterization as an aggregation-prone inactive protein.^7^ The only potentially functional polymerase in the patient is thus derived from the other allele containing the novel F197S substitution. We found that F197S has reduced exonuclease and polymerase activities compared with WT POLγA, which can explain the patient phenotype.

Of the over 30 disease-associated mutations located in the exonuclease domain (Human DNA Polymerase Gamma Mutation Database, tools.niehs.nih.gov/polg/), only a few have been shown to be associated with increased mtDNA point mutations.^18^ F197S is uniquely located in motif I, adjacent to the D198 residue that is essential for 3′-5′ exonuclease activity.^19^ As expected from the location and strong conservation, F197S showed severely reduced 3′-5′ exonuclease activity. Nonetheless, this appears to be sufficient to deal with nucleotide misincorporation because only a very modest increase in mtDNA point mutations was observed in the patient.

We instead propose that the pathogenic molecular defect lies in reduced polymerase activity. That a mutation in the exonuclease domain can affect polymerase activity is unexpected but not unprecedented^20,21^ and may be due to an imbalance between the polymerase and exonuclease activities. The inefficient polymerase activity explains the patient's disease phenotype and presence of mtDNA rearrangements. Recently, we were able to reconstitute mtDNA deletion formation in vitro and could demonstrate that replication stalling during L-strand synthesis causes copy-choice recombination and deletion formation.^9^ Most reported deletions in patients are found in the major arc of mtDNA, a ∼11-kb region that becomes single-stranded during H-strand replication and acts as an ssDNA template during L-strand synthesis. The strong replication stalling by F197S on ssDNA can explain the mtDNA deletions identified in the patient.

We also found mtDNA breakpoints consistent with duplications over the promoter region (mtDNA position 300–1,000 nt). Whether duplications themselves are pathogenic is not clear because no genetic material is lost. Exactly how these duplications arise is not known, but it would be interesting to investigate whether the G-rich conserved sequence block region causes extra problems for polymerases to bypass. It is intriguing to speculate that replication stalling, in combination with the high transcription activity in this area (both light and heavy strand promoters are located here), causes the duplications. In future, we will try to identify specific sequences that are hotspots for polymerase stalling to elucidate the formation of duplications/deletions further.

More than 300 pathogenic *POLG* mutations have been reported, but the pathogenicity can be difficult to establish. Here, we applied in vitro assays to investigate the functional properties of specific POLγ mutants, which in combination with clinical investigation and detailed analysis of mtDNA rearrangements by means of genome sequencing demonstrated not only pathogenicity but also deepens our understanding of the pathobiology. This approach will be essential in the future to evaluate novel *POLG* variants and already described variants that are not fully characterized with regard to pathogenicity.

## Glossary

dsDNA: double-stranded DNA
ES: exome sequencing
mtDNA: mitochondrial DNA
mtSSB: mitochondrial single-stranded DNA binding protein
PEO: progressive external ophthalmoplegia
ssDNA: single-stranded DNA
WT: wild type
